# Epidemiology, treatment and outcomes of gastroenteropancreatic neuroendocrine neoplasms

**DOI:** 10.1038/s41598-024-81518-4

**Published:** 2024-12-17

**Authors:** Johannes Uhlig, James Nie, Joanna Gibson, Michael Cecchini, Stacey Stein, Jill Lacy, Pamela Kunz, Hyun S. Kim

**Affiliations:** 1 Division of Vascular and Interventional Radiology, Department of Diagnostic Radiology and Nuclear Medicine, University of Maryland, Baltimore, USA; 2 Department of Clinical and Interventional Radiology, University Medical Center Goettingen, Goettingen, Germany; 3 Section of Interventional Radiology, Department of Radiology and Biomedical Imaging, Yale School of Medicine, 330 Cedar Street, New Haven, CT 06510 USA; 4 Department of Pathology, Yale School of Medicine, Yale New Haven Hospital, 20 York Street, EP2-610, New Haven, CT 06510 USA; 5 Section of Medical Oncology, Department of Medicine, Yale School of Medicine, 333 Cedar Street, New Haven, CT 06510 USA; 6 Yale Cancer Center, Yale University, 330 Cedar Street, New Haven, CT 06510 USA; 7 Stewart Greenebaum Comprehensive Cancer Center, University of Maryland School of Medicine, Baltimore, MD USA

**Keywords:** Neuroendocrine tumors, Incidence, Survival, Cancer epidemiology, Gastrointestinal cancer, Surgical oncology

## Abstract

To investigate incidence, treatment patterns and outcomes of gastroenteropancreatic neuroendocrine neoplasms (GEP-NEN) in the United States. The 2019 National Cancer Database was searched for adult GEP-NEN patients. Main outcomes included overall and site-specific incidence, treatment patterns, and overall survival (OS). Overall survival was evaluated using averaged Cox regression. 86,324 GEP-NEN patients were included (6.33% of all GEP malignancies). From 2004 to 2016, annual GEP-NEN cases increased (*n* = 4,010 to *n* = 9,379), largely driven by low-stage, low-grade disease. Most patients received surgery, either alone (72.9%) or in combination with systemic therapy (4.9%). Longest overall survival (OS) was evident in patients with low stage and low grade GEP-NEN of the small intestine and rectum (*p* < 0.001). Patients undergoing surgical resection demonstrated longest OS. The addition of systemic therapy was most effective in high stage G3 NEN. Having higher income (*≥*$63,333) and private insurance or Medicare, but not Medicaid, was associated with improved survival. GEP-NEN incidence increases, likely due to improved detection and diagnosis. Treatment patterns have evolved to follow the latest international guidelines and site-specific improvement in survival is noted. In addition to disease specific factors, insurance access and socioeconomic factors emerged as potential targets for improving outcomes.

**Supplementary Information:**

The online version contains supplementary material available at 10.1038/s41598-024-81518-4.

## Introduction

Neuroendocrine neoplasms (NEN) are a heterogenous cancer entity derived from neoplastic transformation of neuroendocrine cells throughout the body with approximately two-thirds arising from the gastroenteropancreatic system^1–3^. Gastroenteropancreatic neuroendocrine neoplasms (GEP-NEN) are predominantly indolent, but there is considerable heterogeneity in their clinical presentation based on site of origin, hormone secretion, disease stage, and histological grade^4–6^.

Since their discovery in 1907, GEP-NEN classification has undergone numerous revisions as data informing their biology has emerged^4^. As of 2019, the World Health Organization (WHO) classifies GEP-NEN by histologic differentiation pattern as well-differentiated neuroendocrine tumor (WDNET) or poorly-differentiated neuroendocrine carcinoma (PDNEC) (Supplemental Table 1; representative pathology in Supplemental Figs. 1 and 2)^3,7^. The vast majority of GEP-WDNET tumors are G1 or G2, while GEP-NEC are G3^7^. The histologic differentiation and WHO classification of grade mirror the distinct molecular differences between GEP-WDNET and GEP-NEC. For instance, pancreatic WDNETs have mutations in DAXX/ATRX, MEN1 or the mTOR pathway, whereas pancreatic NECs have mutations in RB1, TP53 and CDKN2A^8–11^. Recent publications and the WHO have reported on the new category of GEP-WDNET G3 tumors, which are distinct histologically and molecularly from poorly differentiated NEC/G3 cancers^7,12,13^.

Although GEP-NEN are considered rare, several large population-based studies have reported an increasing incidence over the past four decades, possibly attributable to increased use of cross-sectional imaging and endoscopic procedures and more widely adopted classification schemes^1,5,14–16^. In comparison to incidence, only in recent years has survival in GEP-NEN been shown to improve^4,5,16^. Recent approvals of everolimus, sunitinib, and peptide receptor radionuclide therapy (PRRT) may further bolster this trend^1^. To date, there is scarce literature reporting on the treatment and associated outcomes for GEP-NEN. Improved understanding of GEP-NEN is vital as their prevalence increases due to their indolent behavior^17^. In this study, we comprehensively evaluate GEP-NEN regarding contemporary incidence in the United States, current treatment approaches and associated outcomes.

## Materials and methods

### Data source

The 2019 participant user file of the National Cancer Database (NCDB) was queried in March 2020 comprehensively evaluate incidence, treatment patterns, and outcomes of GEP-NEN. The NCDB was established in 1989 as a joint quality improvement project between the Commission on Cancer of the American College of Surgeons and the American Cancer Society. It contains data on over 34 million records from more than 1500 Commission on Cancer–accredited facilities in the United States, making it approximately 3 times larger than the Surveillance, Epidemiology, and End Results (SEER) database^18^. Approximately 70% of newly diagnosed cancer patients are covered by the NCDB annually. Still, the NCDB data are not representative to the general US-population.

This retrospective study was approved by the Yale University School of Medicine ethics committee. Due to the retrospective nature of the study and the evaluated anonymized NCDB data, the Yale University School of Medicine ethics committee waived the need of obtaining informed consent. This study was conducted in accordance to the Declaration of Helsinki in its most recent version.

### Study cohort

Adult patients diagnosed between 2004 and 2016 with GEP-NEN were identified using site codes (160–169, 170–179, 180, 182–189, 199 and 209, and 250–259) and ICD-03 histologic codes (8013, 8150–8153, 8155–8157, 8240–8242, 8244–8246, 8249, and 9091). The following ICD-03 histological codes were excluded: 8243 (goblet cell carcinoid), 8154 (mixed islet-cell/ exocrine adenocarcinoma), and 8574 (adenocarcinoma with neuroendocrine features). We also excluded primary diagnoses of lung cancer, medullary carcinoma of the thyroid, pheochromocytoma, and paraganglioma as well as patients with secondary cancer diagnoses. NEN were further divided in pancreatic NEN and gastrointestinal NEN (GI-NEN). GEP cancers of non-neuroendocrine histologies from the same site codes were queried for epidemiological comparison. The follow-up period ended on January 1st, 2017, after which patients were right-censored. Exclusion criteria were age < 18, AJCC stage 0 disease, and missing data on survival status, metastatic sites, and follow-up time. Socioeconomic variables and tumor factors were evaluated as potential predictors of outcomes. The primary objective of this study was to assess overall survival, defined as the number of months from diagnosis to date of death or censoring.

### Variables

Data collected included patient-level variables (age, sex, race, insurance status, education, residency area, region, comorbidities measured by Charlson-Deyo Comorbidity index), hospital-level variables (academic vs. non-academic), and tumor-level variables (year of diagnosis, tumor location, time from diagnosis to treatment, stage, grade, tumor size, presence and location of metastases. T and N categories were classified by the higher rating when comparing clinical and pathological assessment). To ensure patient anonymity, the NCDB suppressed facility type and location for patients aged under 39 years. First line treatment was stratified by surgical resection of the primary tumor with or without addition of systemic therapy, or systemic therapy without surgical resection. In the NCDB, “systemic therapy” refers to, but does not distinguish between various cytotoxic chemotherapies, sunitinib, or everolimus. Other therapies, such as somatostatin analogues, were either not captured or recorded in limited capacity and were thus not included in treatment analysis. NCBD does not provide Ki-67 proliferative index or mitotic rates, but does report tumor grades as which for this study were summarized as “G1, G2” (well differentiated; moderately differentiated; intermediate differentiation) and “G3” (poorly differentiated; undifferentiated; anaplastic). For the majority of statistical analyses, G1 and G2 tumors were analyzed together, as these could not be reliably distinguished according to the current WHO classification system.

### Statistical analyses

Data were analyzed in March 2020. Continuous variables were compared using the Wilcoxon rank sum test and categorical variables were compared using the χ2 test. Overall survival was initially assessed using the Kaplan-Meier methods and compared using log-rank analysis. Covariates with *p* < 0.10 in univariate analysis were considered for inclusion into the multivariable Cox proportional regression model. The proportional hazards assumption was visually assessed plotting Schoenfeld residuals. Due to evident violations of the proportional hazards assumption, weighted Cox regression models were used for all survival models, implementing Prentice weights, censoring correction and robust variance estimation^19^. For statistical analyses, GEP-NEN tumor locations were partially combined.

A priori, a statistical multiplicative interaction test between tumor grade, tumor stage, and treatment was planned to evaluate varying treatment effectiveness with two-way and three-way interaction terms in the multivariable model. All analyses were performed using R, version 3.4.3 (R Core Development Team, Vienna, Austria), and RStudio, version 1.1.414 (R Studio Inc, Boston, MA). An α level of 0.05 was chosen to indicate statistical significance and all provided P values are 2-sided. Hazard ratios are reported with 95% confidence intervals (95% CI).

## Results

### Patient population

**Fig. 1:**
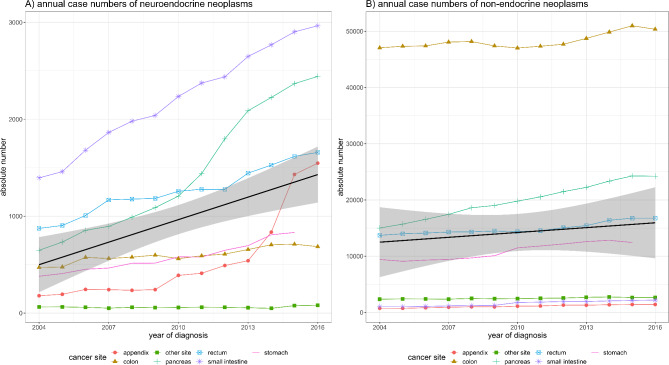
Annual case numbers for gastroenteropancreatic neuroendocrine neoplasms and gastroenteropancreatic non-endocrine neoplasms stratified by location. Overall mean increase provided as black line.

A total of *n* = 86,324 patients with GEP-NEN fulfilled the inclusion criteria (Table 1). Patients were diagnosed at a median age of 58 years (interquartile range [IQR], 50–68 years), with *n* = 42,026 male (48.7%) and *n* = 44,298 female (51.3%) patients. GEP-NEN comprised 6.3% of all cancers of the GEP system diagnosed during this time period (total *n* = 1,363,843). The absolute number of annual new cases of GEP-NEN increased by 133.9% from 2004 to 2016, compared to 9.3% for GEP cancers of non-neuroendocrine histology (Fig. 1). In 2004, GEP-NEN contributed 4.3% of all GEP cancers, increasing to 8.8% by 2016. Compared to gastrointestinal neoplasms of non-neuroendocrine histology, GEP-NEN patients were more likely to be diagnosed at a younger age (median age of 58 vs. 66 years; *p* < 0.0001), be female (51.3% vs. 47.5%; *p* < 0.0001), be African American (17.3% vs. 12.5%; *p* < 0.0001), have private insurance (52.8% vs. 36.8%; *p* < 0.001), belong to higher income brackets (*p* < 0.001), and have fewer comorbidities (*p* < 0.0001). Patient characteristics are summarized in Table 1.

**Table 1:** Baseline characteristics of included patients. Provided p-values assess overall differences in variable distribution in the GEP-NEN and Non-GEP group.

	GEP-NEN No. 86,324	GEP Non-NEN histology No. 1,277,519	*P*-value
Age	58.0 (50.0–68.0)	66.0 (56.0–77.0)	< 0.0001
Gender			< 0.0001
Female	44,298 (51.3%)	607,407 (47.5%)	
Male	42,026 (48.7%)	670,112 (52.5%)	
Race			< 0.0001
African American	14,910 (17.3%)	159,452 (12.5%)	
Caucasian	66,296 (76.8%)	1,044,744 (81.8%)	
Other race	5,118 (5.9%)	73,323 (5.7%)	
Insurance status			< 0.0001
Not Insured	2,963 (3.4%)	49,021 (3.8%)	
Private Insurance/Managed Care	45,610 (52.8%)	470,504 (36.8%)	
Medicaid	5,783 (6.7%)	76,406 (6.0%)	
Medicare	29,021 (33.6%)	642,023 (50.3%)	
Other Government	1,101 (1.3%)	13,838 (1.1%)	
Insurance Status Unknown	1,846 (2.1%)	25,727 (2.0%)	
Median household income for residence area			< 0.0001
< $40,227	14,755 (17.1%)	224,072 (17.5%)	
$40,227 − 50,353	16,407 (19.0%)	254,979 (20.0%)	
$50,354 − 63,332	18,129 (21.0%)	262,621 (20.6%)	
>=$63,333	29,009 (33.6%)	387,078 (30.3%)	
Missing	8,024 (9.3%)	148,769 (11.6%)	
Residency area			< 0.0001
Metropolitan area	72,630 (84.1%)	1,047,748 (82.0%)	
Urban area	8,407 (9.7%)	140,476 (11.0%)	
Rural area/NA	5,287 (6.1%)	89,295 (7.0%)	
Comorbidities [Charlson Score]			< 0.0001
0	65,969 (76.4%)	902,392 (70.6%)	
1	14,850 (17.2%)	266,289 (20.8%)	
2	3,627 (4.2%)	73,341 (5.7%)	
>=3	1,878 (2.2%)	35,497 (2.8%)	
Time diagnosis to treatment			< 0.0001
Median (IQR)	0.0 (0.0–34.0)	15.0 (1.0–31.0)	
Missing	13,087 (15.2%)	206,357 (16.2%)	
Tumor location			< 0.0001
Appendix	6,890 (8%)	14,202 (1.1%)	
Ascending colon	1,318 (1.5%)	121,981 (9.5%)	
Cecum	3,384 (3.9%)	132,012 (10.3%)	
Descending colon	186 (0.2%)	35,385 (2.8%)	
Pancreas	18,774 (21.7%)	258,220 (20.2%)	
Rectosigmoid junction	916 (1.1%)	71,385 (5.6%)	
Rectum	16,370 (19.0%)	194,173 (15.2%)	
Sigmoid colon	1,388 (1.6%)	166,258 (13.0%)	
Small intestine	28,750 (33.3%)	20,578 (1.6%)	
Stomach	6,877 (8.0%)	130,309 (10.2%)	
Transverse colon	588 (0.7%)	100,331 (7.9%)	
Other site	793 (0.9%)	32,685 (2.6%)	
Stage			< 0.0001
Stage I	22,879 (26.5%)	235,430 (18.4%)	
Stage II	8,233 (9.5%)	302,688 (23.7%)	
Stage III	11,746 (13.6%)	289,876 (22.7%)	
Stage IV	15,651 (18.1%)	349,759 (27.4%)	
Unknown stage	27,815 (32.2%)	99,766 (7.8%)	
Tumor grade			< 0.0001
Grade I	37,750 (43.7%)	99,509 (7.8%)	
Grade II	7,726 (9.0%)	613,904 (48.1%)	
Grade III	6,502 (7.5%)	251,212 (19.7%)	
Grade IV	0 (0.0%)	21,820 (1.7%)	
Grade unknown	34,346 (39.8%)	291,074 (22.8%)	
Tumor size [mm]			< 0.0001
Median (IQR)	17.0 (8.0–32.0)	40.0 (29.0–58.0)	
Missing	26,063 (30.2%)	368,155 (28.8%)	
Metastases			< 0.0001
Brain metastasis	103 (0.1%)	2,449 (0.2%)	
Bone metastasis	761 (0.9%)	12,402 (1.0%)	
Liver metastasis	8,348 (9.7%)	126,344 (9.9%)	
Lung metastasis	722 (0.8%)	37,159 (2.9%)	
Missing	39,002 (45.2%)	653,607 (51.2%)	
Treatment			< 0.0001
Systemic alone	5,478 (6.3%)	217,071 (17.0%)	
No surgery or systemic	13,707 (15.9%)	195,336 (15.3%)	
Surgery w/o systemic	62,945 (72.9%)	458,791 (35.9%)	
Surgery + systemic	4,194 (4.9%)	406,321 (31.8%)	
Treatment facility type			< 0.0001
Academic center	31,687 (36.7%)	416,946 (32.6%)	
Non-academic center	46,529 (53.9%)	823,750 (64.5%)	
Facility type suppressed for age 0–39 years	8,108 (9.4%)	36,823 (2.9%)	
Treatment facility location			< 0.0001
East North-Central	14,320 (16.6%)	221,923 (17.4%)	
East South-Central	5,313 (6.2%)	86,810 (6.8%)	
Middle Atlantic	11,765 (13.6%)	196,914 (15.4%)	
Mountain	3,323 (3.8%)	50,141 (3.9%)	
New England	4,508 (5.2%)	70,528 (5.5%)	
Pacific	8,620 (10.0%)	140,164 (11.0%)	
South Atlantic	17,183 (19.9%)	267,197 (20.9%)	
West North-Central	6,298 (7.3%)	94,024 (7.4%)	
West South-Central	6,886 (8.0%)	112,995 (8.8%)	
Facility location suppressed for age 0–39 years	8,108 (9.4%)	36,823 (2.9%)	

## Disease characteristics

**Fig. 2:**
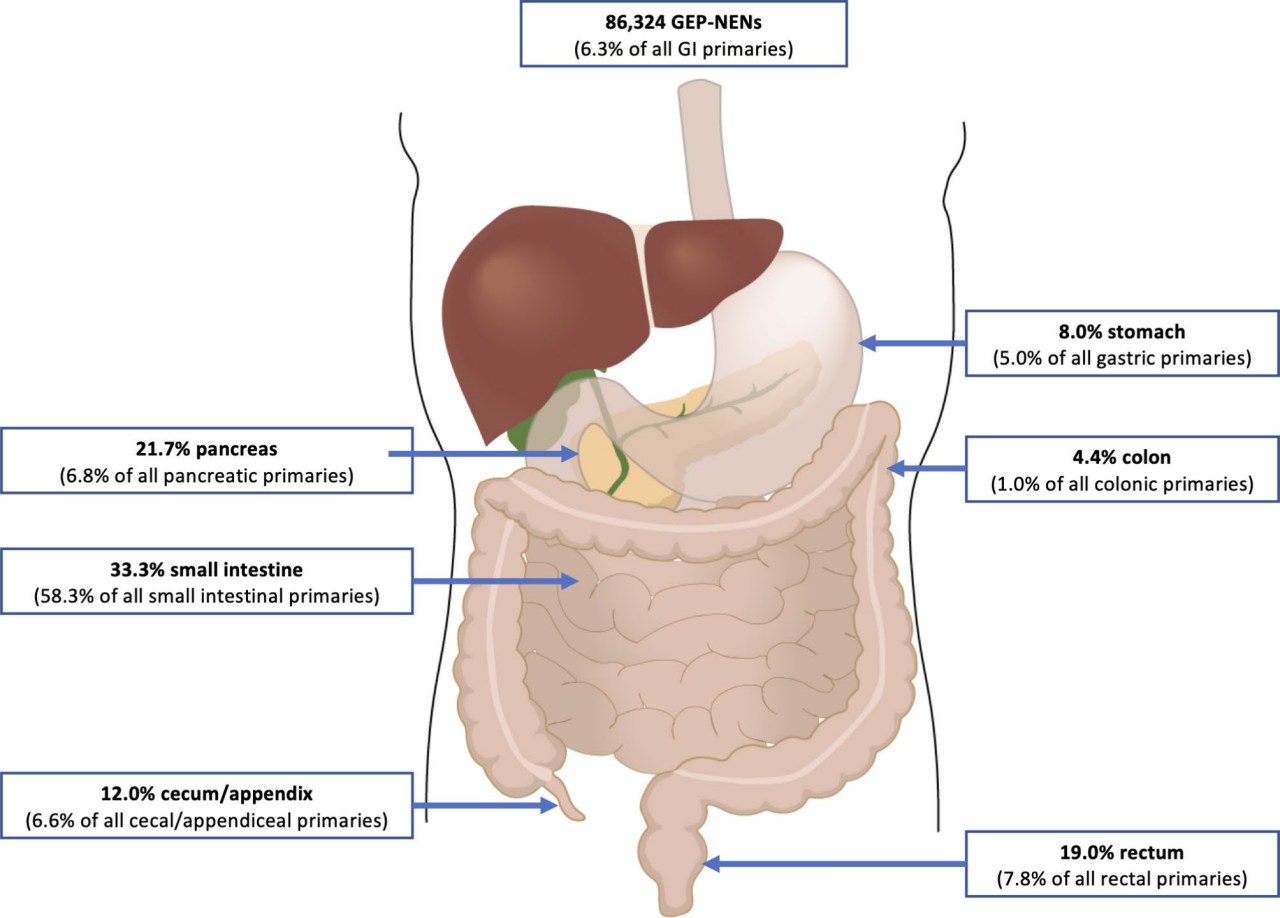
Neuroendocrine neoplasm incidence according to primary location of the gastroenteropancreatic system.

The highest proportion of GEP-NEN originated in the small intestine (33.3%) and lowest in the descending colon (Fig. 2). GEP-NEN patients more commonly were smaller in size (median size 17.0 mm vs. 40.0 mm) and of lower grade compared to GEP non-NEN histologies (*p* < 0.001): 87.5% of GEP-NEN with known grades were G1/G2 (60.2% patients with known grade). 18.1% GEP-NEN patients had stage IV disease at diagnosis, compared to 27.4% for GEP non-neuroendocrine histologies (*p* < 0.001). Among patients with known GEP-NEN grade and stage (*n* = 41,766), a cross tabulation is provided in Supplemental Table 2. The increase in GEP-NEN cases over the study period was driven by low-stage, low-grade disease across most primary sites; pancreatic and small intestine NEN exhibited an increase in high-stage disease (Supplemental Figs. 3 & 4). Notably, the number of NEN with unknown AJCC stage abruptly decreased in 2010 across all strata. Supplemental Fig. 5 summarizes geographic differences in annual GEP-NEN case numbers and stage distribution in the United States.

## Treatment trends

Patients with GEP-NENs were more likely to be treated at an academic center than non-neuroendocrine GI patients (36.7% vs. 32.6%). Most GEP-NEN patients were treated with surgery, either alone (72.9%) or in combination with systemic therapy (4.9%). Only 6.3% of GEP-NEN patients received systemic therapy without surgery and 15.9% of GEP-NEN patients received best supportive care. Stratification by cancer site, stage, and grade revealed that during the study period, utilization of systemic therapy increased for G3 NEN irrespective of site or stage (Supplemental Fig. 6). For GEP-NEN, the mainstay of treatment was surgical resection across all disease stage/strata with additional use of systemic therapy for G3 disease. Use of systemic therapy in G1/2 GI-NEN disease declined during the study period. For high stage, G3 GI-NEN, use of surgery without systemic therapy declined 49% in favor of systemic therapy alone. For pancreatic NEN, surgical resection alone was most often utilized in low-stage G1/2 disease, and alone or with systemic therapy in low stage, G3 disease. For high-stage, G3 pancreatic NEN, most patients received systemic therapy alone by 2016 – the proportion receiving surgery alone and best supportive care declined.

### Survival analyses

**Fig. 3:**
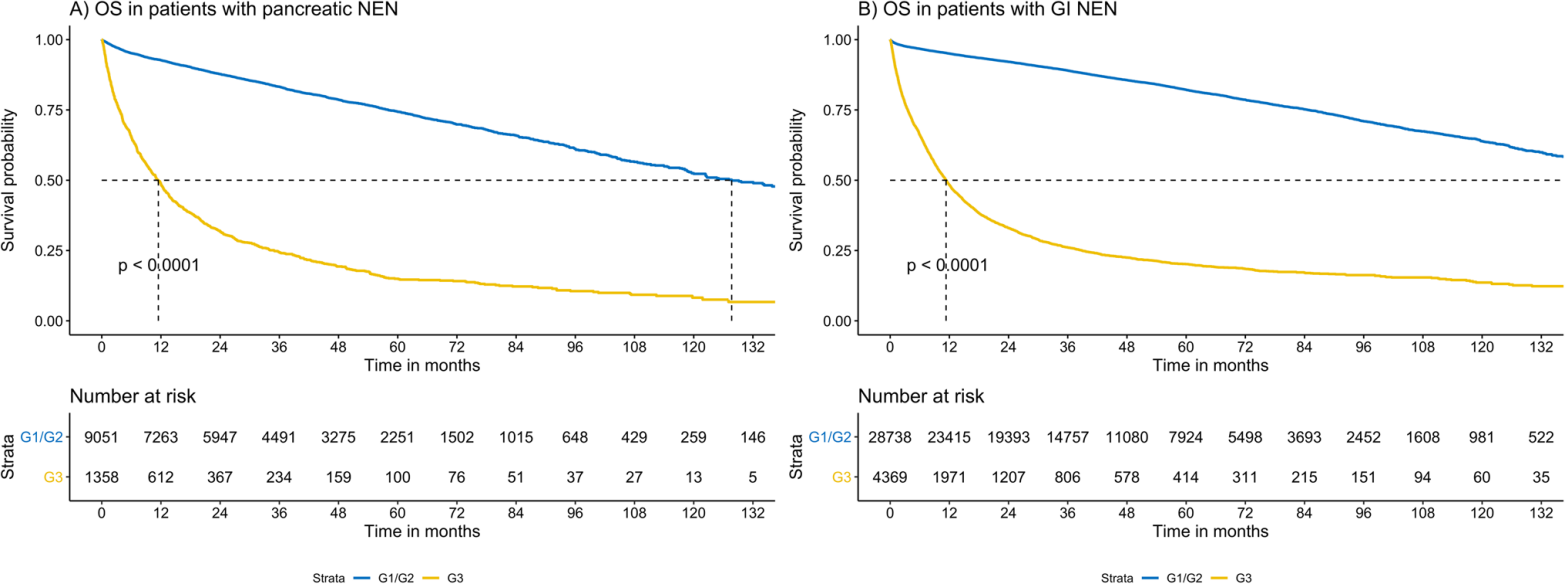
Kaplan-Meier plots showing shorter OS for Grade 3 disease across GI and pancreatic NEN. Grade 1 and Grade 2 tumors were not classified using current WHO criteria (mitotic count and Ki67 proliferative index) given NCBD limitations. Plots were limit to 132 months due to low subsequent event rates.

**Fig. 4:**
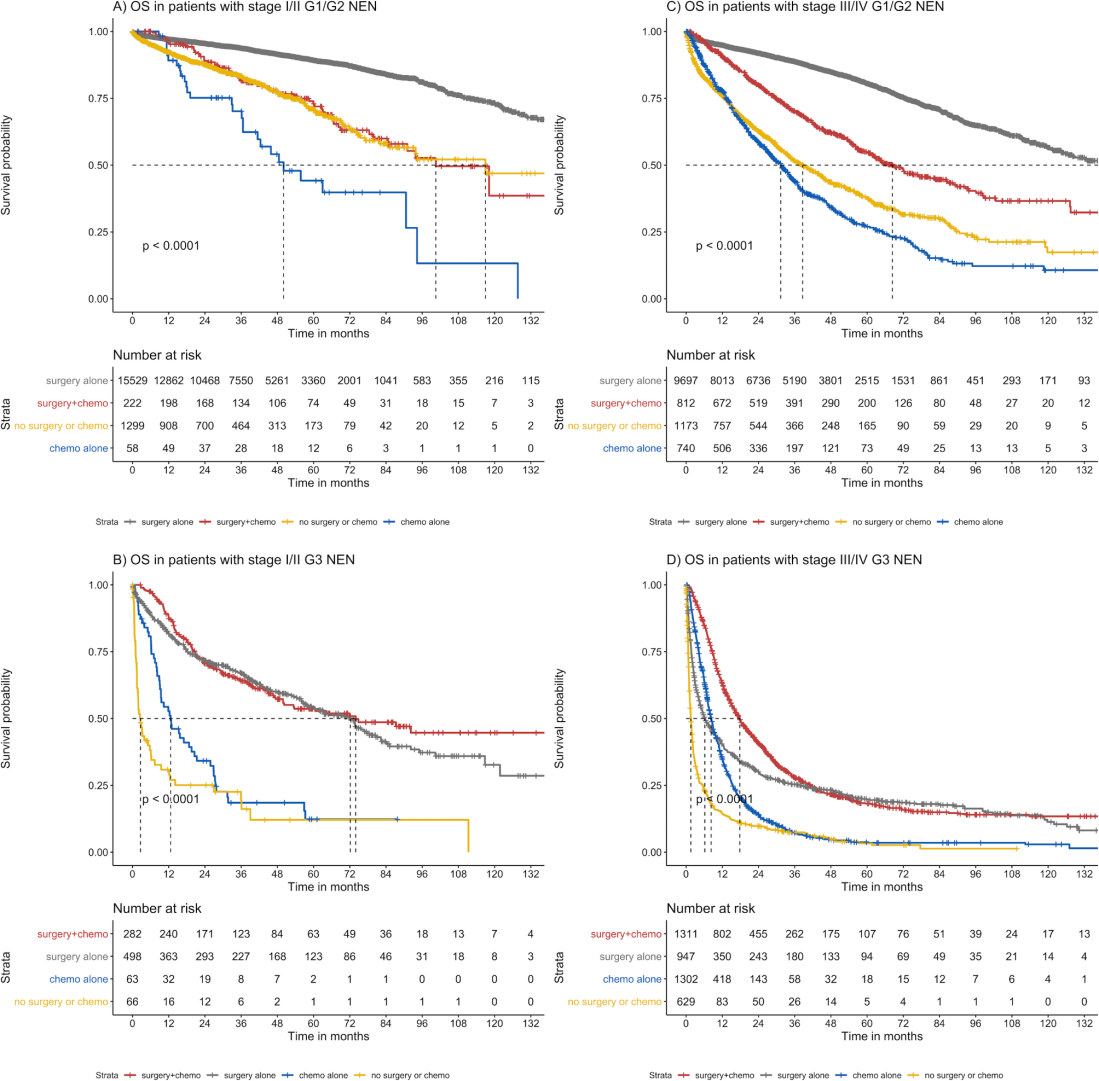
Overall survival of GEP-NEN patients stratified by stage and treatment. Across all strata, patients undergoing surgical resection demonstrated favorable outcomes, with longest OS reported in patients with low-stage and low-grade GEP-NEN. Patients with GEP-NEN benefited from systemic therapy over best supportive care only in Grade 3 disease.

Overall survival was assessed in a cohort of *n* = 43,516 GEP-NEN patients with known grade, follow-up time and survival status. Median follow in this cohort was 44.1 months (IQR: 23.7–71.9 months). For statistical modelling, tumor size was not considered due to missing data. On multivariable analyses, OS was associated with grade, stage, location, and treatment (Table 2). Shorter OS was evident for patients with G3 vs. G1/G2 GEP-NEN (HR = 3.15 (95% CI: 2.81–3.52), *p* < 0.001) (Fig. 3). Efficacy of surgical resection and systemic therapy varied by stage and grade (Fig. 4): across all strata, patients undergoing surgical resection demonstrated favorable outcomes, with longest OS reported in patients with stage 1/2 G1/2 GI-NEN (median OS > 156 months). In G3 disease, the combination of surgery and systemic therapy demonstrated longer OS versus surgery alone. Factors associated with improved survival on multivariate analysis included having higher income (*≥*$63,333) having private insurance, Medicare, or other government insurance, and receiving treatment at an academic center. Of note, having Medicaid was not associated with improved survival over being uninsured.

**Table 2:** Univariate and multivariable Cox regression models evaluating overall survival, using weighted regression with Prentice weights, censoring correction and robust variance estimation to account for violations of the proportional hazards assumption. Variables with *p* < 0.1 in univariate models were considered for inclusion in the final multivariable model, and retained if multivariable *p* < 0.05. * facility location not included in the multivariable model due to collinearity with facility type.

Variable	Levels	Univariate HR (95% CI)	Multivariable HR (95% CI)
Age	[18.0,90.0]	1.04 (1.02–1.05), *p* < 0.001	1.04 (1.03–1.04), *p* < 0.001
Gender	Female	1 (reference)	1 (reference)
	Male	1.07 (0.97–1.18), *p* = 0.149	1.09 (1-1.19), *p* = 0.042
Race	African American	1 (reference)	-
	Caucasian	1.14 (0.99–1.32), *p* = 0.066	-
	others	0.73 (0.59–0.9), *p* = 0.004	-
Proportion of patients without highschool diploma	>=17.6%	1 (reference)	-
	10.9–17.5%	0.7 (0.44–1.09), *p* = 0.113	-
	6.3–10.8%	1.14 (0.86–1.5), *p* = 0.356	-
	< 6.3%	0.74 (0.62–0.89), *p* = 0.001	-
Urban	metropolitan area	1 (reference)	-
	urban area	1.99 (1.2–3.3), *p* = 0.007	-
	rural area/NA	1.18 (0.92–1.51), *p* = 0.185	-
Insurance	Not Insured	1 (reference)	1 (reference)
	Private Insurance/Managed Care	0.58 (0.49–0.69), *p* < 0.001	0.61 (0.51–0.72), *p* < 0.001
	Medicaid	0.85 (0.68–1.06), *p* = 0.154	0.84 (0.67–1.05), *p* = 0.132
	Medicare	1.62 (1.37–1.9), *p* < 0.001	0.79 (0.66–0.95), *p* = 0.013
	Other Government	0.67 (0.47–0.94), *p* = 0.022	0.65 (0.47–0.89), *p* = 0.008
	Insurance Status Unknown	0.99 (0.73–1.34), *p* = 0.953	0.9 (0.67–1.21), *p* = 0.487
Income	< $40,227	1 (reference)	1 (reference)
	$40,227 − 50,353	0.94 (0.78–1.13), *p* = 0.51	0.95 (0.82–1.11), *p* = 0.513
	$50,354 − 63,332	0.88 (0.72–1.07), *p* = 0.196	0.86 (0.73–1.02), *p* = 0.075
	>=$63,333	0.73 (0.61–0.87), *p* < 0.001	0.79 (0.68–0.91), *p* = 0.002
Comorbidities (Charlson Deyo Comorbidity Index)	0	1 (reference)	1 (reference)
	1	1.6 (1.35–1.89), *p* < 0.001	1.34 (1.17–1.54), *p* < 0.001
	2	2.19 (1.89–2.53), *p* < 0.001	1.59 (1.38–1.85), *p* < 0.001
	>=3	2.95 (2.4–3.63), *p* < 0.001	2.44 (2.01–2.95), *p* < 0.001
Cancer grade	G1/G2	1 (reference)	1 (reference)
	G3	5.53 (4.96–6.16), *p* < 0.001	3.18 (2.85–3.55), *p* < 0.001
Stage	Stage I	1 (reference)	1 (reference)
	Stage II	2.17 (1.87–2.51), *p* < 0.001	1.45 (1.23–1.69), *p* < 0.001
	Stage III	2.11 (1.82–2.45), *p* < 0.001	1.43 (1.21–1.68), *p* < 0.001
	Stage IV	7.48 (6.6–8.47), *p* < 0.001	3.98 (3.43–4.62), *p* < 0.001
	Unknown stage	2.18 (1.88–2.52), *p* < 0.001	1.69 (1.44–1.99), *p* < 0.001
Cancer site	Appendix	1 (reference)	1 (reference)
	Colon	3.7 (2.92–4.69), *p* < 0.001	1.22 (0.97–1.52), *p* = 0.084
	Other site	0.16 (0.02–1.04), *p* = 0.055	0.05 (0.01–0.31), *p* = 0.001
	Pancreas	2.02 (1.64–2.48), *p* < 0.001	1.11 (0.89–1.37), *p* = 0.362
	Rectum	0.98 (0.74–1.29), *p* = 0.881	0.77 (0.59–1.01), *p* = 0.059
	Small intestine	1.61 (1.31–1.99), *p* < 0.001	0.95 (0.77–1.18), *p* = 0.651
Treatment	Chemo alone	1 (reference)	1 (reference)
	No surgery or chemo	0.44 (0.37–0.52), *p* < 0.001	0.82 (0.7–0.96), *p* = 0.011
	Surgery w/o chemo	0.14 (0.12–0.17), *p* < 0.001	0.4 (0.34–0.46), *p* < 0.001
	Surgery + chemo	0.43 (0.35–0.52), *p* < 0.001	0.5 (0.42–0.59), *p* < 0.001
Year of cancer diagnosis	[2004.0,2015.0]	0.94 (0.93–0.95), *p* < 0.001	0.97 (0.96–0.98), *p* < 0.001
Facility type	Academic center	1 (reference)	1 (reference)
	Facility type suppressed for age 0–39 years	0.45 (0.37–0.54), *p* < 0.001	1.59 (1.22–2.06), *p* < 0.001
	Non-academic center	1.23 (1.11–1.36), *p* < 0.001	1.19 (1.09–1.3), *p* < 0.001
Facility location	East North Central	1 (reference)	-
	East South Central	1.15 (0.74–1.77), *p* = 0.541	-
	facility location suppressed for age 0–39 years	0.38 (0.31–0.47), *p* < 0.001	-
	Middle Atlantic	0.9 (0.78–1.03), *p* = 0.117	-
	Mountain	0.8 (0.65–0.98), *p* = 0.033	-
	New England	0.82 (0.61–1.1), *p* = 0.192	-
	Pacific	0.82 (0.69–0.98), *p* = 0.026	-
	South Atlantic	1.08 (0.94–1.25), *p* = 0.271	-
	West North Central	1.01 (0.83–1.22), *p* = 0.948	-
	West South Central	0.93 (0.8–1.08), *p* = 0.343	-

## Discussion

To our knowledge, this is the first study to examine epidemiology, treatment, and outcomes of GEP-NEN in a national cohort. Using contemporary US data, we demonstrated that between 2004 and 2016, the annual number of GEP-NEN cases more than doubled, growing disproportionately in comparison to non-neuroendocrine GEP histologies. These findings mirror the magnitude of increasing incidence of GEP-NENs reported in recent North American population-based studies^5,14,16,20^. The greatest increases seen in GEP-NEN sites were the cecum/appendix (5.4-fold), pancreas (3.9-fold), and stomach (2.4-fold). For non-neuroendocrine GEP neoplasms, only the rates of small intestine (2.2-fold) and pancreatic diagnoses (1.6-fold) increased substantially. While increased use of cross-sectional imaging and endoscopy has significantly outpaced growth of non-neuroendocrine GEP cancers, it has significantly increased detection of earlier stage disease GEP-NEN^5,17,21^. Within our cohort, low-grade and non-metastatic disease contributed to the majority of the increase in annual GEP-NEN diagnoses (Supplemental Figs. 1 & 2). A portion of the increase in low-grade disease can be attributed to wider adoption of classification systems, reflected in the decline in cases with unknown grade and unknown stage after 2010^22^.

Interestingly, our sample showed increased detection of both low and high-stage pancreatic NENs. Increased incidental detection has made it such that non-functioning pancreatic NENs now make up the majority of new pancreatic NEN diagnoses^23^. Earlier diagnosis of these non-functioning tumors, of which > 50% have been found to exhibit malignant potential, may have contributed to the improved 5-year survival noted in our sample compared to a SEER analysis from a decade ago (65% vs. < 40%).^16^ The advent of new targeted therapies such as sunitinib or everolimus in advanced disease may have also contributed to this trend. The 5-year survival of colonic and gastric primaries in our cohort are similar to a SEER study from a decade ago, possibly due to the lack of increase in low grade or low stage disease at both sites^16^.

In our cohort, treatment effectiveness varied by stage and grade: across all strata, patients treated with surgical resection demonstrated longest overall survival. This is in line with international guidelines that recommend surgical resection as the mainstay of treatment for low grade local or locoregional GEP-NEN^24^. Present guidelines differ in their recommendations for cytotoxic chemotherapy: the European Society for Medical Oncology recommends chemotherapy for advanced pancreatic NENs and high grade NENs of any site, whereas the National Comprehensive Cancer Network (NCCN) recommends chemotherapy for advanced pancreatic NENs only^24,25^. Within NCCN, there is disagreement on whether to offer chemotherapy for advanced GI-NEN given the low response rates and lack of PFS benefit^25^. Our data show that addition of systemic therapy, which largely corresponds to chemotherapy in NCDB (but not somatostatin analogues), was most effective in G3 stage III and IV disease, with use increasing across all G3 disease over time. Use of systemic therapy in low grade GI-NEN declined during the study period, but approximately 5% of patients with low grade, high stage disease still received systemic therapy in 2016. For a subset of aggressive, low-grade GI-NENs, some NCCN panelists make provisions for using cytotoxic chemotherapy when no other options are available^24–26^. Use of cytotoxic chemotherapy in this subset of patients may explain the worse outcomes in G1/G2 disease for systemic therapy across all stages of GEP-NEN (Fig. 4).

While the combination of surgery and systemic therapy was associated with the best OS for high-stage, G3 GEP-NENs, studies in recent years suggest differential response based on histology^27,28^. Response to platinum-based chemotherapy has been noted to be substantially greater for patients with Ki-67 proliferation index of > 55%, which most often corresponds to NEC, whereas surgery has been associated with better survival for G3 GEP-NETs than for GEP-NEC^27–29^.

In our cohort, privately insured patients made up a higher proportion of GEP-NEN compared to other GI cancers, perhaps reflective of the younger age at diagnosis and improved access to diagnostic imaging for detection of disease. We found treatment at an academic center to be associated with better outcomes, suggestive of the importance of integrating multi-disciplinary expertise in managing GEP-NENs^30^. Given the delays in obtaining a GEP-NEN diagnosis, difficulty in finding a treatment center with sufficient expertise, and the chronic course of most GEP-NENs, having proper access to care and sufficient financial resources can drastically alter disease prognosis^30–32^. Insured GEP-NEN patients have been shown to be significantly more likely to receive somatostatin analogues and less likely to undergo emergency surgery for tumor resection^31^. While we report survival benefit for privately insured and Medicare patients, the surprising finding that Medicaid patients have worse outcomes than uninsured patients in multivariate analysis shows that socioeconomic factors beyond access to care are important for survival. This corroborates findings from a universal healthcare system study: a Canadian study found that NEN patients with low socioeconomic status had worse outcomes despite similar stage at diagnosis and similar treatment^33^. In multivariable analysis, a recent SEER study that suggests that the racial disparities in GEP-NEN survival are decreasing and that the differences now seen are driven largely by socioeconomic and psychosocial factors^33,34^. We similarly did not find racial differences in survival in multivariate analysis nor did we find survival differences for patients living in rural versus metropolitan areas, in line with another SEER study^35^. Geographically, the West North-Central and West South-Central United States were associated with higher rates of stage III/IV disease, yet survival differences across regions were marginal in univariate analysis.

The strength of this study lies in the detailed assessment of demographic, diagnostic, and treatment trends from a large patient population representative of typical patients treated in the United States. Still, this study has several limitations. The NCDB collects hospital-based data, which cannot provide population-wide incidence, with potential limitations to the generalizability of our findings. Although the NCDB covers approximately 70% of newly diagnosed cancer cases in the USA annually, it is not necessarily representative of the US-population, unlike the SEER database (which collects data from representative geographic regions and subpopulations). However, the increasing GEP-NEN cases mirror the findings from other population-based studies^5,14–16^. Additionally, studies have shown that survival parameters are comparable between the NCDB and SEER database, for example in patients with CRC, while patients demographics may vary slightly^35^. Further, the comparison of NEN to all non-NEN histologies made in this study fail to reflect on the heterogeneity of histological subtypes of specific cancer sites, for example of the appendix or pancreas.

NCDB also does not report tumor specific variables such as Ki-67 or mitotic rate and contains some use of discontinued histological terms, making classification of G1 and G2 disease unreliable given evolving classification systems during the study period. Thus, G1 and G2 disease were combined for this analysis. Nevertheless, we are confident that the G3 classification in our cohort is reliable, as the terms used for classification have been associated with G3 tumors throughout the study period.

The NCDB does not report cancer-specific survival nor does it capture specific chemotherapies, targeted molecular agents, or somatostatin analogues so treatment efficacy cannot be compared. Few data exist to compare efficacy of systemic GEP-NEN therapies, but recent meta-analysis suggests that combination of systemic therapies, particularly those involving somatostatin analogues, may be most effective^36^. In patients with unresectable liver metastases, improvement in patient selection and investigation into the potential of somatostatin receptor analogues and targeted agents to sensitize the effects of locoregional therapy may further improve health-related quality of life for patients with treatment-refractory disease^37–39^.

## Conclusion

Using the NCDB, we demonstrated that absolute number of GEP-NEN cases has continued to rise from 2004 to 2016, possibly due to increased diagnosis of early stage disease. Survival is dictated not only by disease specific factors, but also access to care and socioeconomic status. Promisingly, real world treatment has evolved to follow evidence driven guidelines. The recent technological improvements in GEP-NEN therapeutics, refinement of disease classification, and growing awareness of the social barriers involved in this disease may help drive improved outcomes in the years to come.

## Electronic supplementary material

Below is the link to the electronic supplementary material.

Supplementary Material 1

## Data Availability

The data that support the findings of this study are available from the American College of Surgery but restrictions apply to the availability of these data, which were used under license for the current study, and so are not publicly available. Data are however available from the corresponding authors upon reasonable request and with permission of the American College of Surgery.
