# Supplementary material for: Epidemiology, treatment and outcomes of gastroenteropancreatic neuroendocrine neoplasms
Source: Sci Rep. 2024 Dec 17;14:30536. doi: 10.1038/s41598-024-81518-4 (PMC11652651; doi:10.1038/s41598-024-81518-4)
Supplement: Supplementary file 1 — Supplementary Material 1 [file 41598_2024_81518_MOESM1_ESM.docx]

**Supplemental Figures**

**
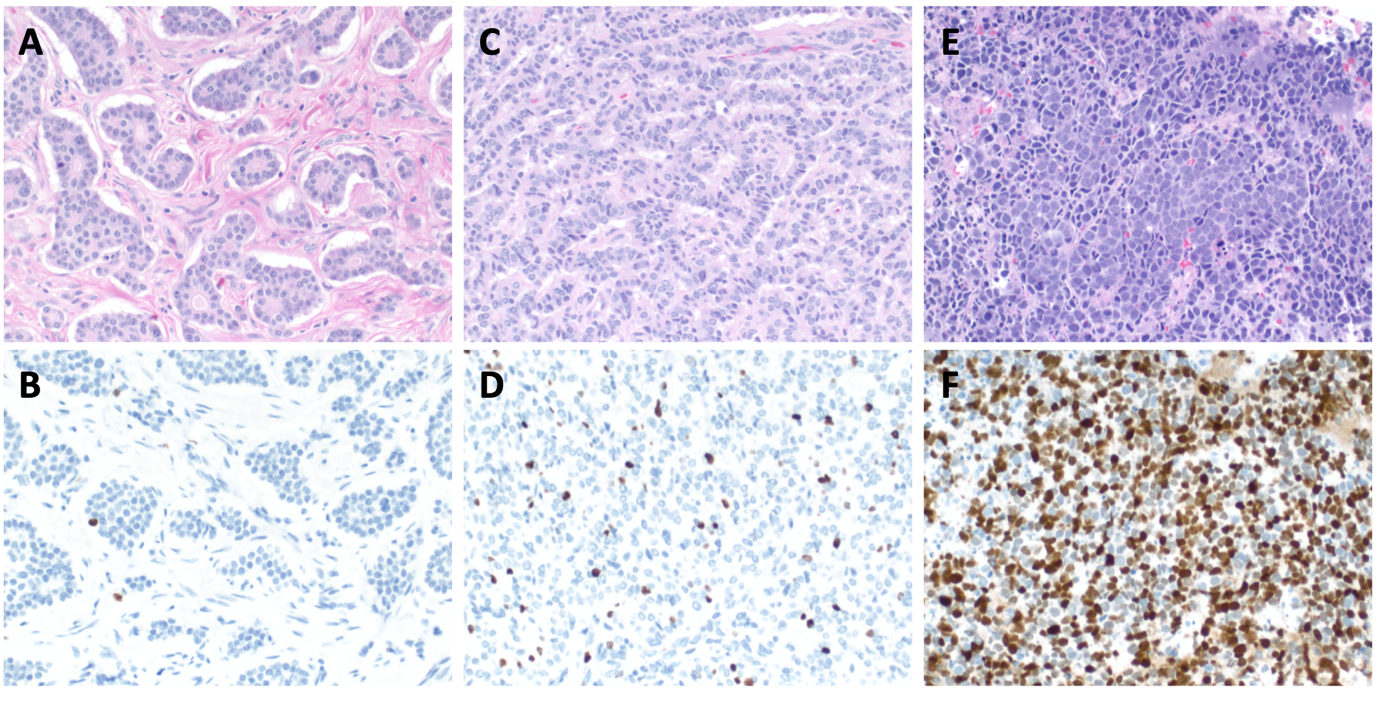
**

**Figure 1:** H&E of small bowel WDNET (A), WHO Grade 1, with 0.4% Ki-67 proliferative index (B). H&E of pancreas WDNET (C), WHO Grade 2, with 9% Ki-67 proliferative index (D). Poorly differentiated NEC (E), WHO Grade 3, with >90% Ki-67 proliferative index (F). All H&E images are 200x magnification. Ki-67 proliferative index determined using manual count method (B&D) or visual estimation (F).

**
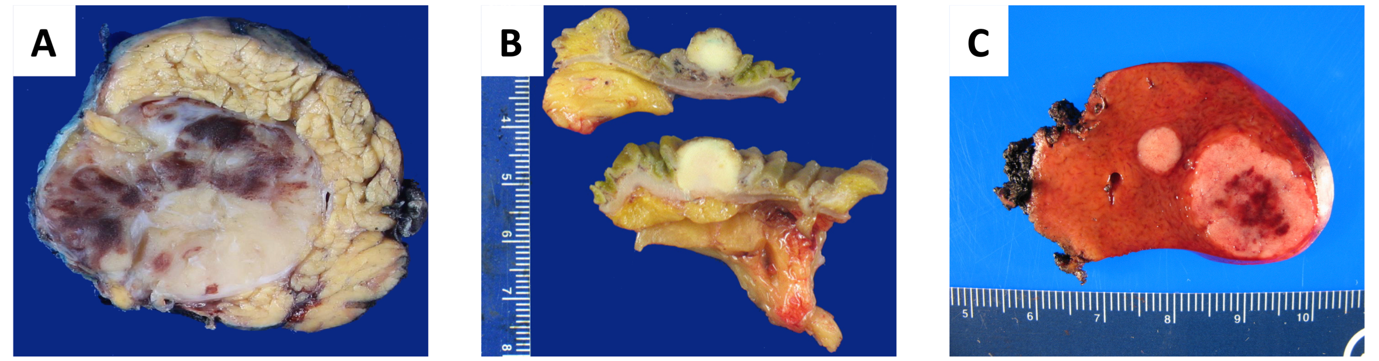
**

**Figure 2:** Gross pathology of WDNET arising in pancreas (A) and small bowel (B) and WDNET metastasis to liver (C).

**
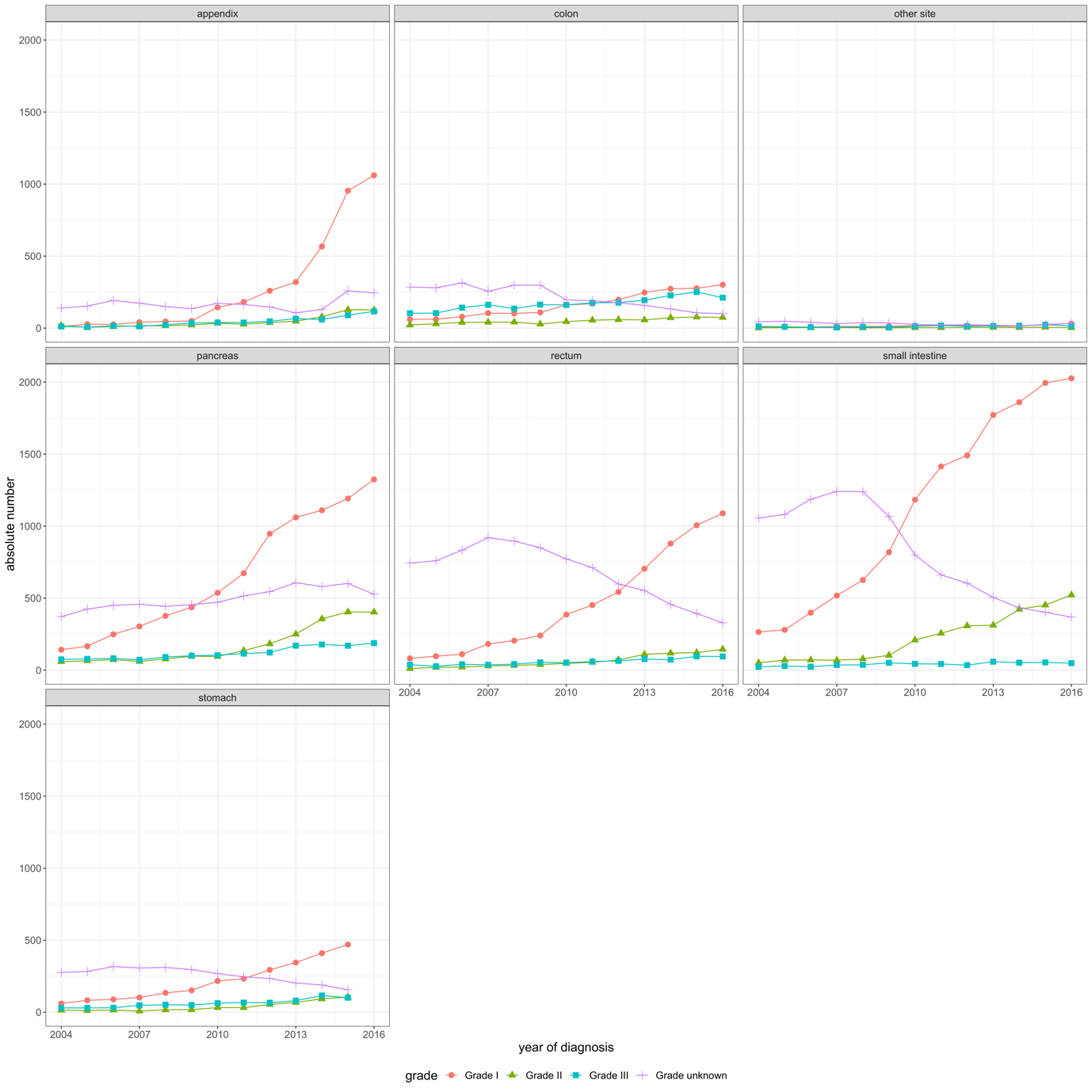
**

**Supplemental Figure 3:** Number of newly diagnosed cases of GEP-NEN by site and grade. Increases in Grade 1 disease largely drive the overall increasing incidence in GEP-NEN.

**
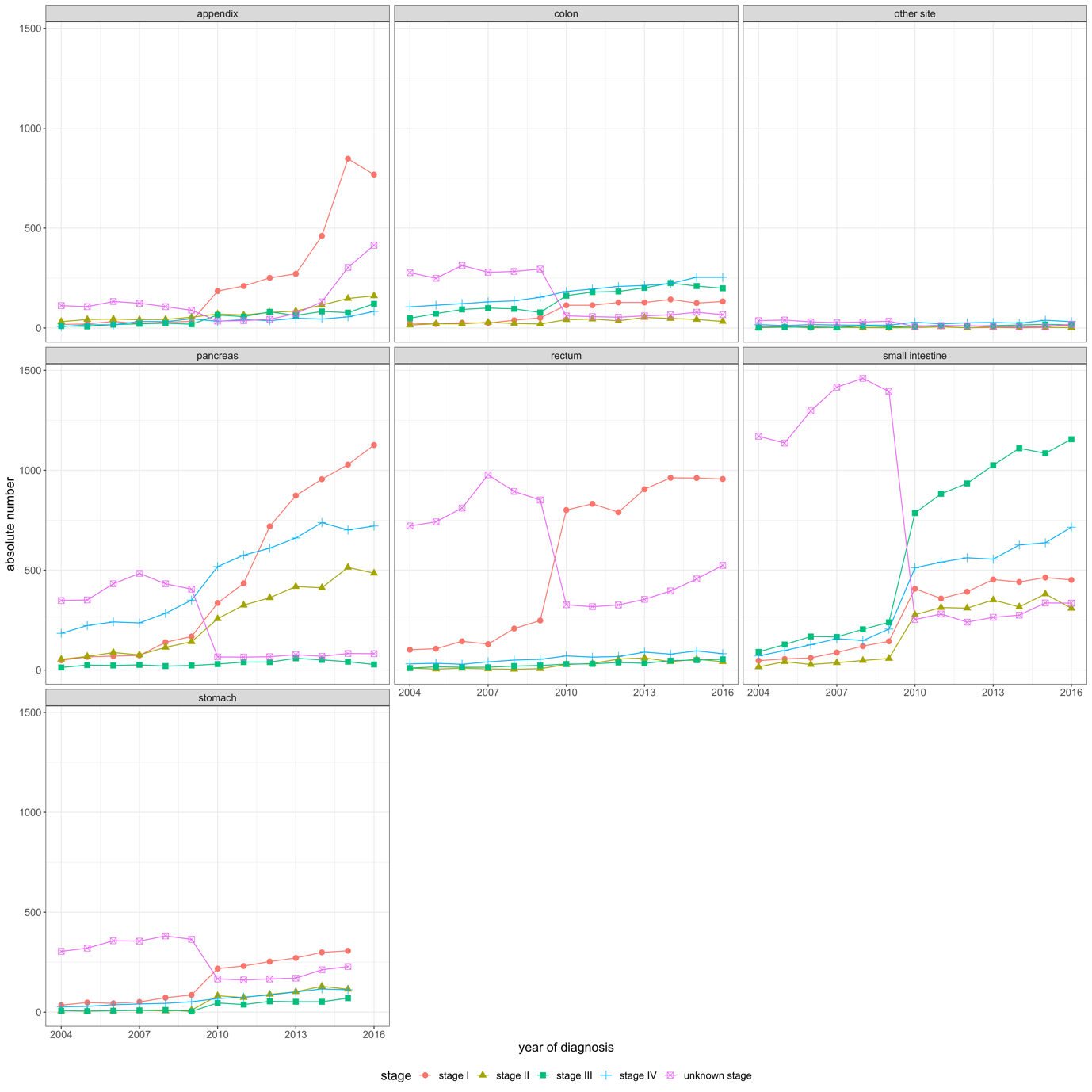
**

**Supplemental Figure 4:** Number of newly diagnosed cases of GEP-NEN by site and stage. The largest increase is seen in low stage disease across most sites. Pancreatic and small intestine NEN exhibited an increase in high-stage disease. Sharp decline in unknown stage correlates with the introduction of the 2010 WHO classification system.

**
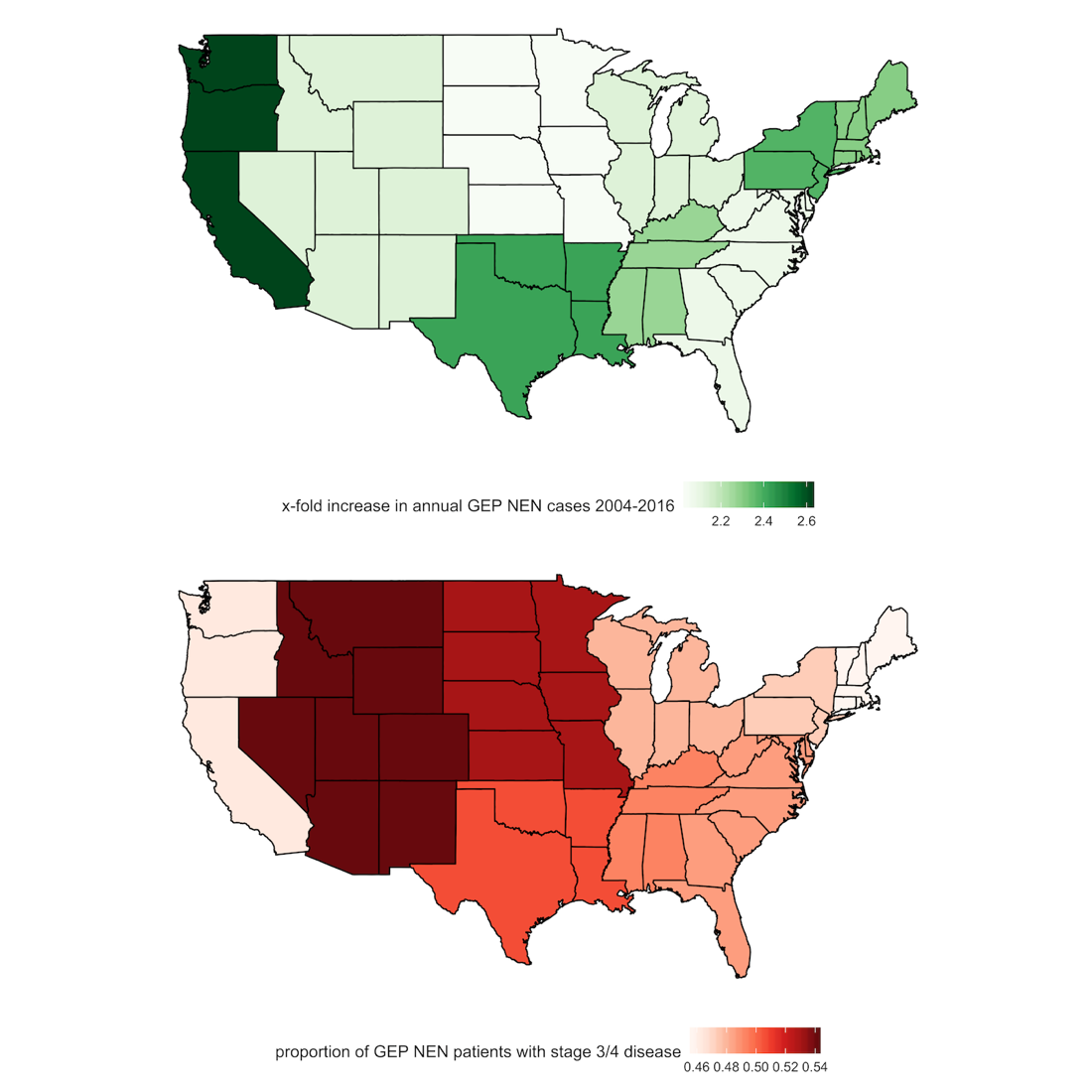
**

**Supplemental Figure 5:** The proportional change of newly diagnosed GEP-NEN cases by geography, overall and high stage disease.The West Central Northern and West Central Southern regions of the United States exhibited the greatest fold increase in GEP-NEN diagnoses and have the highest proportion of higher stage disease. Map created using R, version 3.4.3 (R Core Development Team, Vienna, Austria), and RStudio, version 1.1.414 (R Studio Inc, Boston, MA)

**
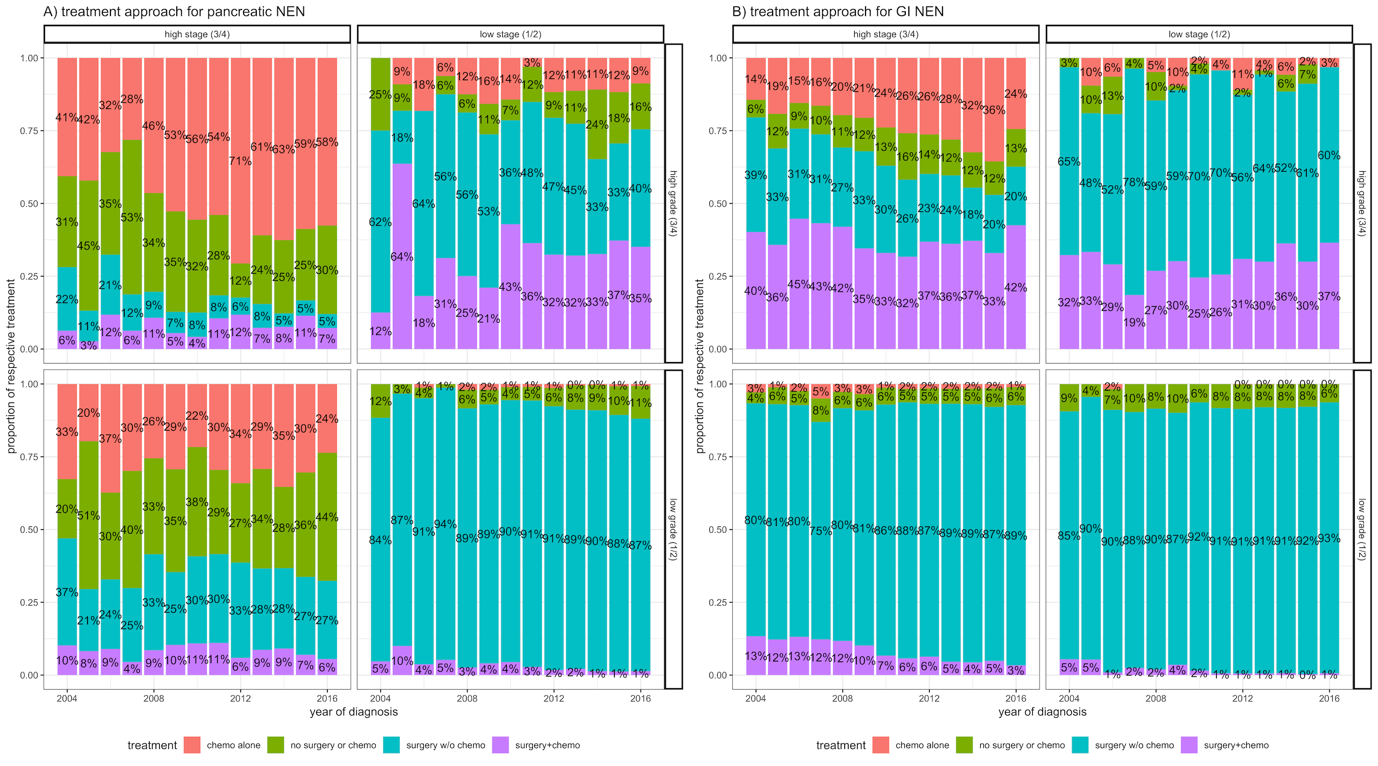
**

**Supplemental Figure 6:** Treatment patterns of GEP-NENs by grade and stage over time. The mainstay of treatment was surgical resection, with additional use of systemic therapy for Grade 3 disease. Utilization of systemic therapy increased for Grade 3 GEP-NEN irrespective of site or stage.

**Supplemental Table 1:** 2019 WHO classification of gastroenteropancreatic neuroendocrine neoplasms. Due to numerous changes in the classification system during the study period, Grade 1 and Grade 2 disease were combined for analyses.^7^

| Classification/Grade | Mitotic Index | Ki-67 Proliferation Index (%) | Molecular signature |
| --- | --- | --- | --- |
| Well-differentiated neuroendocrine tumor | | | |
| Grade 1 | <2 | <3 | Mutations in DAXX/ATRX, MEN1, or mTOR pathway |
| Grade 2 | 2-20 | 3-20 |  |
| Grade 3 | >20 | >20 |  |
| Poorly-differentiated neuroendocrine carcinoma | | | |
| Grade 3 | >20 | >20 | Mutations in RB1, TP53, CDKN2A |

**Supplemental Table 2:** Cross tabulation of stage and grade for pancreatic and GI NEN

| **Pancreatic NEN** | **high grade (3/4)** | **low grade (1/2)** |
| --- | --- | --- |
| high stage (3/4) | 960 | 2153 |
| low stage (1/2) | 383 | 7304 |

| **GI NEN** | **high grade (3/4)** | **low grade (1/2)** |
| --- | --- | --- |
| high stage (3/4) | 3828 | 12625 |
| low stage (1/2) | 661 | 13852 |
